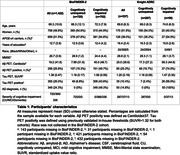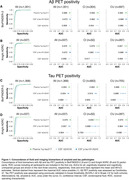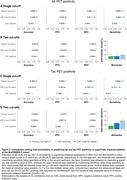# A highly accurate blood test for Alzheimer’s disease pathology has performance equivalent or superior to clinically used CSF tests

**DOI:** 10.1002/alz.089139

**Published:** 2025-01-09

**Authors:** Gemma Salvadó, Nicolas R. Barthélemy, Suzanne E. Schindler, Yingxin He, Shorena Janelidze, Lyduine E. Collij, Benjamin A. Saef, Rachel L. Henson, Charles D. Chen, Brian A. Gordon, Yan Li, Renaud La Joie, Tammie L.S. Benzinger, John C. Morris, Niklas Mattsson‐Carlgren, Sebastian Palmqvist, Rik Ossenkoppele, Gil D. Rabinovici, Erik Stomrud, Randall J. Bateman, Oskar Hansson

**Affiliations:** ^1^ Clinical Memory Research Unit, Department of Clinical Sciences, Lund University, Lund Sweden; ^2^ Washington University in St. Louis School of Medicine, St. Louis, MO USA; ^3^ The Tracy Family SILQ Center, St. Louis, MO USA; ^4^ The Tracy Family SILQ Center, Saint Louis, MO USA; ^5^ Knight Alzheimer Disease Research Center, St. Louis, MO USA; ^6^ Washington University School of Medicine, Saint Louis, MO USA; ^7^ Department of Radiology and Nuclear Medicine, Vrije Universiteit Amsterdam, Amsterdam University Medical Center, location VUmc, Amsterdam Netherlands; ^8^ Amsterdam Neuroscience, Brain Imaging, Amsterdam Netherlands; ^9^ Lund University, Lund Sweden; ^10^ Washington University in St. Louis, School of Medicine, St. Louis, MO USA; ^11^ Department of Radiology, Washington University School of Medicine, Saint Louis, MO USA; ^12^ University of California, San Francisco, San Francisco, CA USA; ^13^ Washington University in St. Louis, St. Louis, MO USA; ^14^ Memory Clinic, Skåne University Hospital, Malmö Sweden; ^15^ Clinical Memory Research Unit, Department of Clinical Sciences Malmö, Faculty of Medicine, Lund University, Lund Sweden; ^16^ Wallenberg Center for Molecular Medicine, Lund University, Lund Sweden; ^17^ Alzheimer Center Amsterdam, Neurology, Vrije Universiteit Amsterdam, Amsterdam UMC location VUmc, Amsterdam Netherlands; ^18^ Department of Radiology and Biomedical Imaging, University of California, San Francisco, San Francisco, CA USA; ^19^ Department of Neurology, Memory and Aging Center, University of California San Francisco, San Francisco, CA USA; ^20^ Clinical Memory Research Unit, Department of Clinical Sciences, Lund University, Malmö Sweden; ^21^ SILQ Center for Neurodegenerative Biology, St. Louis, MO USA; ^22^ Knight Alzheimer's Disease Research Center, St. Louis, MO USA

## Abstract

**Background:**

With the emergence of Alzheimer's disease (AD) disease‐modifying therapies, identifying patients who could benefit from these treatments becomes critical. We evaluated whether a precise blood test could perform as well as established cerebrospinal fluid (CSF) tests in detecting amyloid‐β (Aβ) plaques and tau tangles.

**Method:**

Plasma %p‐tau217 (ratio of phosporylated‐tau217 to non‐phosphorylated mid‐region tau) was analyzed by mass spectrometry in the Swedish BioFINDER‐2 cohort (n=1,422) and the US Knight ADRC cohort (n=337, Table 1). Matched CSF samples were analyzed with clinically used and FDA‐approved automated immunoassays for Aβ42/40 and p‐tau181/Aβ42. The primary and secondary outcomes were detection of brain Aβ or tau pathology (Braak I‐IV), respectively, using PET imaging as the reference standard. Main analyses were focused on individuals with cognitive impairment (mild cognitive impairment and mild dementia), which is the target population for available disease‐modifying treatments. We assessed accuracy, positive and negative predictive values, and sensitivity of each biomarker, using cut‐offs derived at 90% specificity for a one cut‐off approach. We also tested a two cut‐off approach using cut‐offs derived at 95% specificity and 95% sensitivity, classifying participants as positive (above higher cut‐off), negative (below lower cut‐off) or intermediate (between these two cut‐offs).

**Result:**

Plasma %p‐tau217 was clinically equivalent to FDA‐approved CSF tests in classifying Aβ PET status, with an area‐under‐the‐curve (AUC) for both cohorts between 0.95‐0.97 (Figure 1A‐B). Plasma %p‐tau217 was generally superior to CSF tests in classification of tau‐PET with AUCs of 0.95‐0.98 (Figure 1C‐D). In cognitively impaired sub‐cohorts (BioFINDER‐2: n=720; Knight ADRC: n=50), plasma %p‐tau217 had an accuracy, positive predictive value and negative predictive value of 89‐90% for Aβ PET (Figure 2A) and 87‐88% for tau‐PET status (Figure 2C), which was clinically equivalent to CSF tests. These statistics further improved up to 95% using a two cut‐off approach (Figure 2B‐2D, respectively).

**Conclusion:**

Blood plasma %p‐tau217 demonstrated performance clinically equivalent or superior to clinically used FDA‐approved CSF tests in the detection of AD pathology. Use of high performance blood tests in clinical practice can improve access to accurate AD diagnosis and AD‐specific treatments.